# Author Correction: Mitochondria-derived peptide SHLP2 regulates energy homeostasis through the activation of hypothalamic neurons

**DOI:** 10.1038/s41467-023-40832-7

**Published:** 2023-08-17

**Authors:** Seul Ki Kim, Le Trung Tran, Cherl NamKoong, Hyung Jin Choi, Hye Jin Chun, Yong-ho Lee, MyungHyun Cheon, ChiHye Chung, Junmo Hwang, Hyun-Ho Lim, Dong Min Shin, Yun-Hee Choi, Ki Woo Kim

**Affiliations:** 1 Division of Physiology, Department of Oral Biology, Yonsei University College of Dentistry, Seoul, 03722 Korea; 2 Department of Applied Life Science, BK21 FOUR, Yonsei University College of Dentistry, Seoul, 03722 Korea; 3 Neuroscience Research Institute, Seoul National University College of Medicine, Seoul, 03080 Korea; 4 Department of Biomedical Sciences, Seoul National University College of Medicine, Seoul, 03080 Korea; 5 Department of Internal Medicine, Yonsei University College of Medicine, Seoul, 03722 Korea; 6 Department of Biological Sciences, Konkuk University, Seoul, 05029 Korea; 7 Neurovascular Unit Research Group, Korea Brain Research Institute (KBRI), Daegu, 41068 Korea

**Keywords:** Feeding behaviour, Neuronal physiology, Obesity

Correction to: *Nature Communications* 10.1038/s41467-023-40082-7, published online 19 July 2023

In this article the funding from ‘the Korea Drug Development Fund, funded by the Ministry of Science and ICT, the Ministry of Trade, Industry, and Energy, and the Ministry of Health and Welfare (HN22C0645, Republic of Korea)’ was omitted. The original article has been corrected.

