# Supplementary material for: Mitochondria-derived peptide SHLP2 regulates energy homeostasis through the activation of hypothalamic neurons
Source: Nat Commun. 2023 Jul 19;14:4321. doi: 10.1038/s41467-023-40082-7 (PMC10356901; doi:10.1038/s41467-023-40082-7)
Supplement: Supplementary file 1 — Supplementary Information [file 41467_2023_40082_MOESM1_ESM.pdf]

Mitochondria-derived peptide SHLP2 regulates energy homeostasis through the activation of hypothalamic neurons

Seul Ki Kim[1,2], Le Trung Tran[1,2], Cherl NamKoong[3], Hyung Jin Choi[3,4], Hye Jin Chun[5], Yong-ho Lee[5], MyungHyun Cheon[6], ChiHye Chung[6], Junmo Hwang[7], Hyun-Ho Lim[7], Dong Min Shin[1,2], Yun-Hee Choi[1], and Ki Woo Kim[1,2 *]

[1]Division of Physiology, Department of Oral Biology, Yonsei University College of Dentistry, Seoul, 03722, Korea

[2]Department of Applied Life Science, BK21 FOUR, Yonsei University College of Dentistry, Seoul, 03722, Korea

[3]Neuroscience Research Institute, Seoul National University College of Medicine, Seoul, 03080, Korea

[4]Department of Biomedical Sciences, Seoul National University College of Medicine, Seoul, 03080, Korea

[5]Department of Internal Medicine, Yonsei University College of Medicine, Seoul, 03722, Korea

[6]Department of Biological Sciences, Konkuk University, Seoul, 05029, Korea

[7]Neurovascular Unit Research Group, Korea Brain Research Institute (KBRI), Daegu, 41068, Korea

[*]Correspondence and reprints, materials should be addressed to:

Ki Woo Kim (kiwoo-kim@yuhs.ac)

SUPPLEMENTARY INFORMATION

Supplementary Figure 1-6

Supplementary Tables 1-3

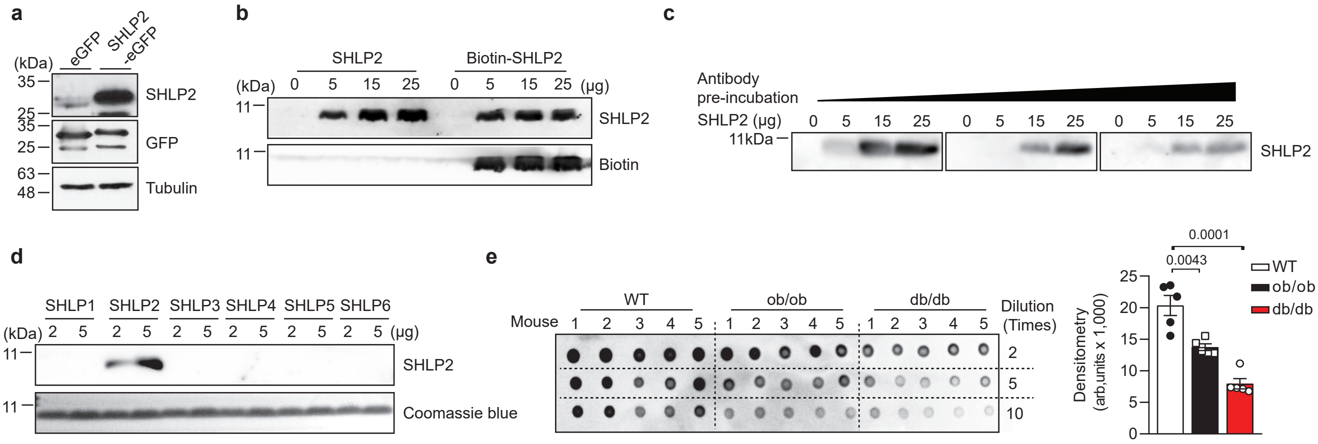

Supplementary Fig. 1: Validation of SHLP2 antibody.

a, Detection of SHLP2 after overexpression of SHLP2-eGFP construction in HEK 293 cells. Western blots were performed using anti-SHLP2, anti-GFP or anti-tubulin antibodies. **b**, Detection of SHLP2 and biotinylated SHLP2 peptides using anti-SHLP2 and anti-biotin antibodies. **c**, Peptide competition assays using SHLP2 peptides. Anti-SHLP2 antibody was incubated in the presence of increasing amounts (1, 5, 15, 25 µg) of each peptide indicated. **d**, Detection of SHLP1 to 6 using anti-SHLP2 antibody. **e**, Dot-blot image (left) and relative blot densitometry (right) detecting plasma SHLP2 levels in WT, *ob/ob* and *db/db* male mice (n = 5 each group). Data shown are representative of three independent experiments with similar results (**a-d**). n indicates the number of biologically independent mice examined. Data were presented as mean ± SEM. Two-tailed Student's t-tests were used. Source data are provided as a Source Data file.

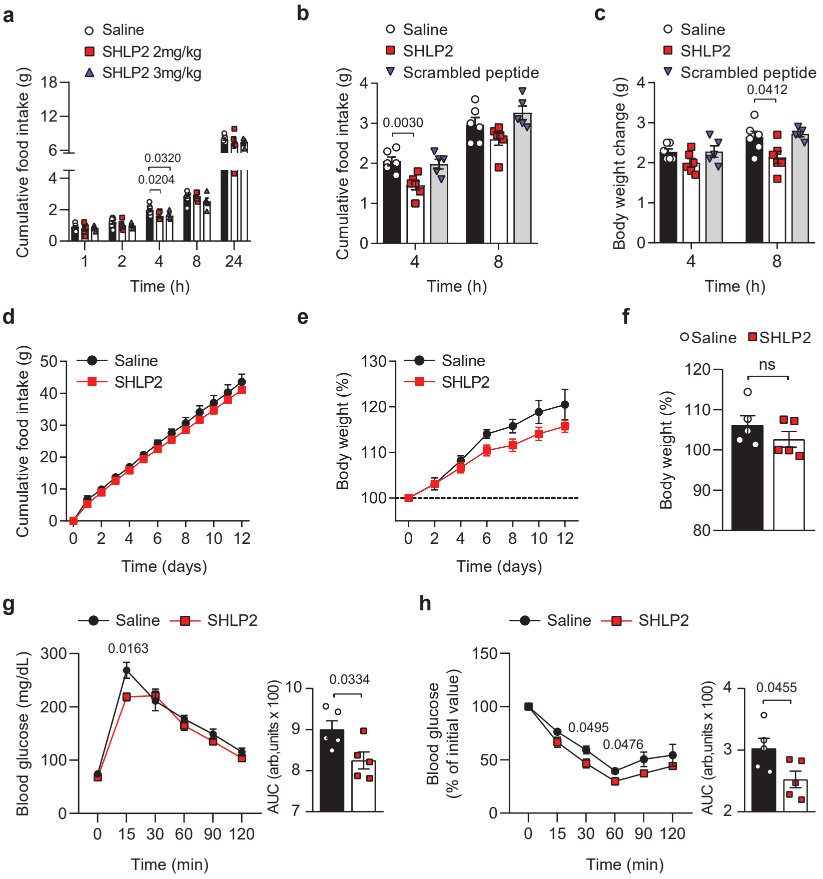

Supplementary Fig. 2: Temporal changes in food intake and body weight, as well as the results of the ITT and GTT, in normal chow-fed mice after an IP injection of SHLP2

a, Cumulative food intake (left) of male mice fed with NC after IP injection of saline or 2, 3mg/kg SHLP2 (*n* = 9 saline, *n* = 5 each SHLP2 group). **b-c,** Cumulative food intake (**b**) and body weight change (**c**) of male mice fed with NC after IP injection of saline or SHLP2 or scrambled peptide following an overnight fasting (*n* = 6 saline, *n* = 6 SHLP2, *n* = 5 scrambled peptide). **d-e**, Cumulative food intake (**d**) and body weight change (% of initial) (**e**) were monitored after daily IP injection of either saline or SHLP2 in male mice (*n* = 3 saline, *n* = 5 SHLP2). **f**, Body weight (% of initial) of male mice fed with NC after daily IP injection of saline or SHLP2 for 3 weeks (*n* = 5 each group). **g-h**, GTT (**g**) and ITT (**h**) were performed in NC-fed male mice after 3 weeks of IP administration of saline or SHLP2 (*n* = 5 each group). *n* indicates the number of biologically independent mice examined. Data were presented as mean ± SEM. Two-tailed Student's t-tests were used. Source data are provided as a Source Data file.

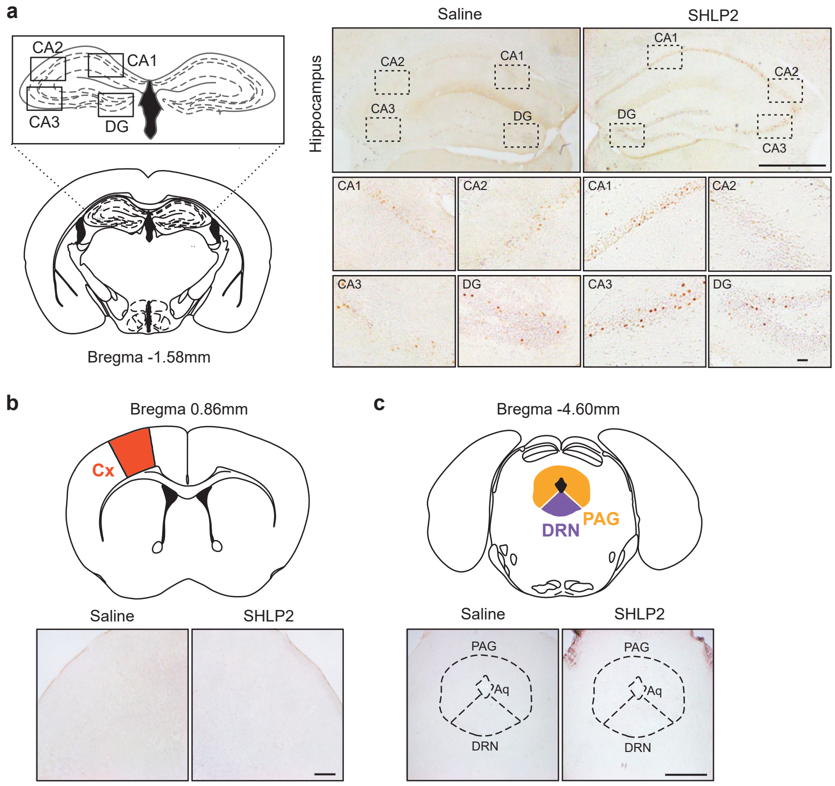

Supplementary Fig. 3: c-Fos activation in different brain regions in response to SHLP2.

a-c, c-Fos immunoactivity after SHLP2 administration (IP) in the hippocampus (**a**)**,** cortex (Cx. **b**), periaqueductal gray (PAG, **c**) and dorsal raphe nucleus (DRN, **c**) (n = 4 each group). CA, cornu amonis. DG, dentate gyrus. Aq, aqueduct. n indicates the number of biologically independent mice examined. Scale bars, 100µm. Source data are provided as a Source Data file.

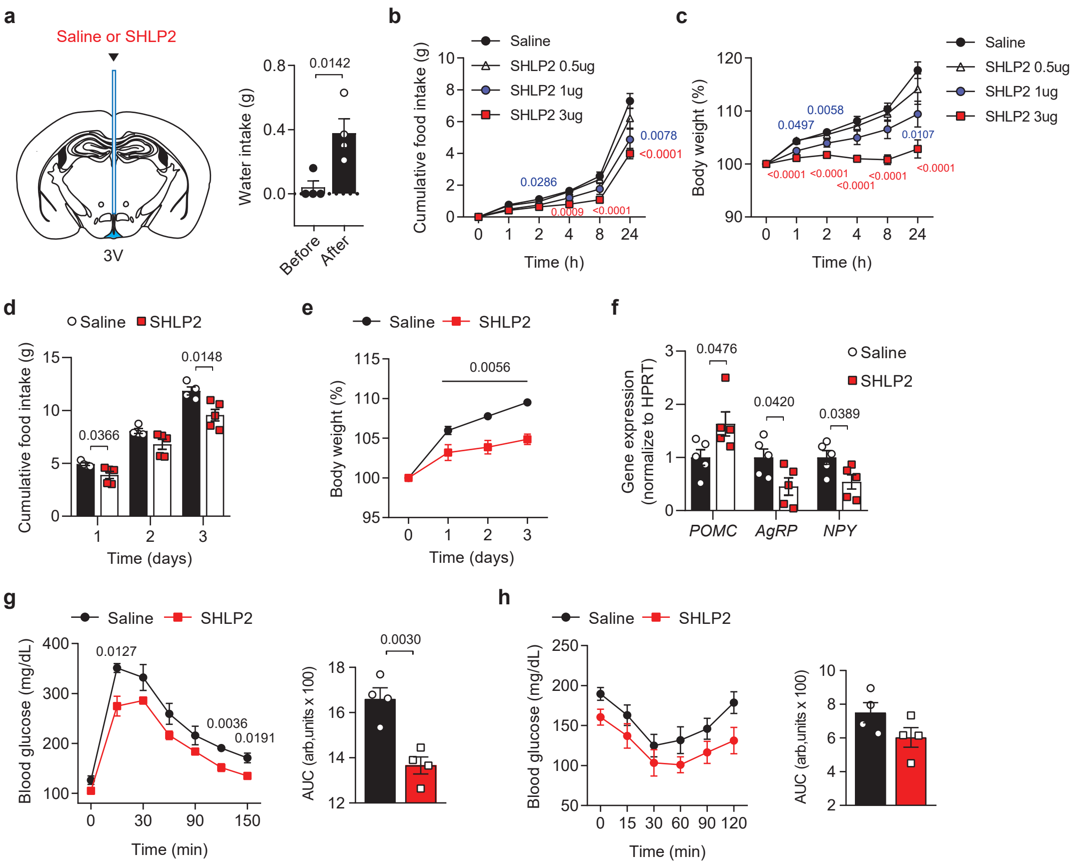

Supplementary Fig. 4: Long-term SHLP2 treatment ameliorates HFD-induced obesity.

a, Schematic for experimental configuration. A guide cannula was implanted to the 3V to deliver saline or SHLP2 (left). Water intake (right) following an acute ICV administration of angiotensin II (*n* = 4 each group). **b-c**, Cumulative food intake (**b**) and body weight (% of initial) (**c**) of male mice fed with NC after ICV injection of saline or 0.5, 1, 3μg SHLP2 (*n* = 10 each group). **d-e**, Cumulative food intake (**d**) and body weight (% of initial) (**e**) of HFD-fed male mice following a single ICV injection of saline or SHLP2 (*n* = 4 saline, *n* = 5 SHLP2). **f**, The *Pomc*, *Agrp* and *Npy* expression in the hypothalamus of male mice fed HFD after one hour of ICV injection of Saline or SHLP2 (*n* = 4 saline, n = 5 SHLP2). **g-h**, GTT (**g**) and ITT (**h**) were performed in HFD-fed male mice after 4 hours of ICV administration of saline or SHLP2 (*n* = 4 each group). *n* indicates the number of biologically independent mice examined. Data were presented as mean ± SEM. Two-tailed Student's t-tests were used. and two-way ANOVA with Bonferroni post-hoc tests were used in figure (**e**). Source data are provided as a Source Data file.

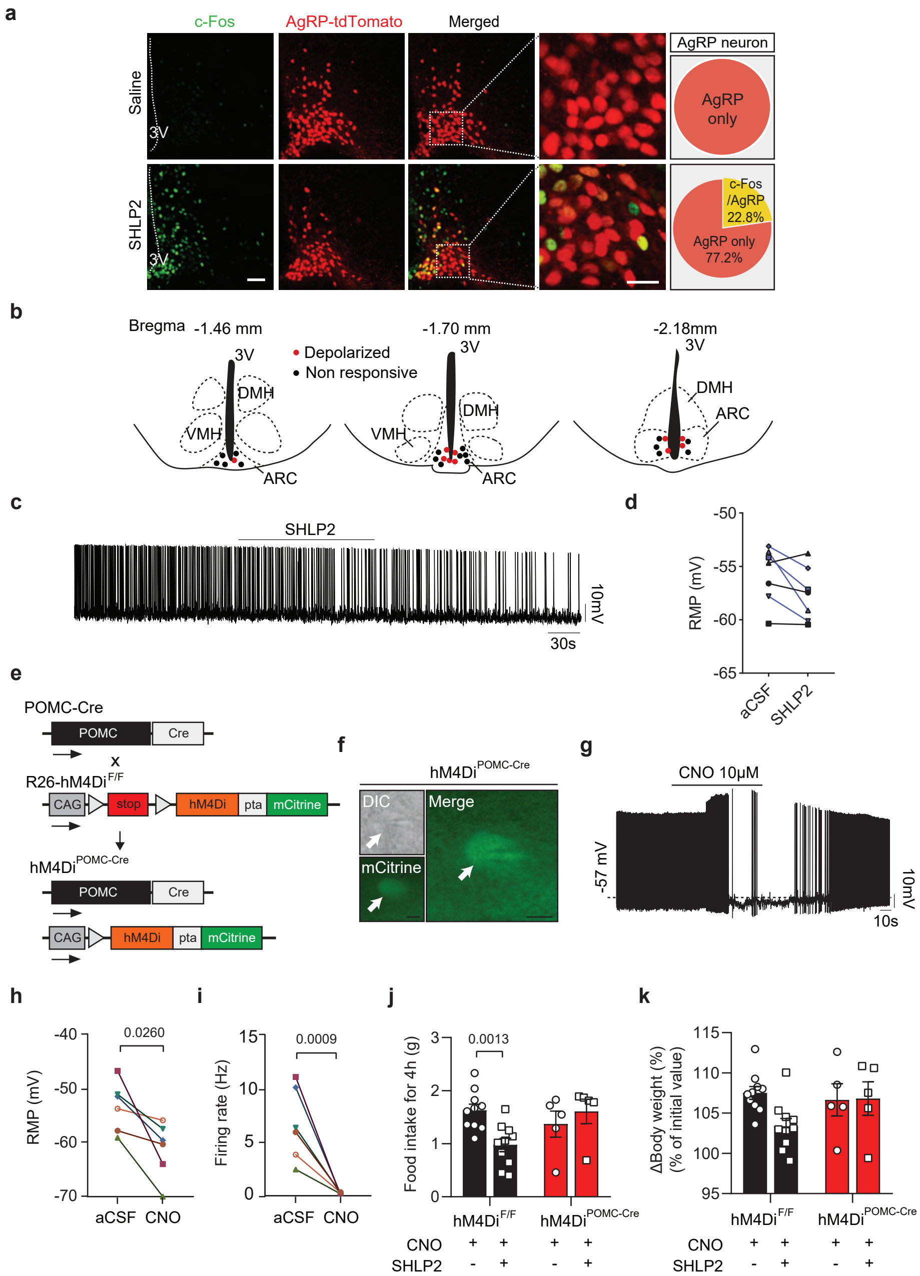
a
c-Fos
AgRP-tdTomato
Merged
AgRP neuron
Saline
SHLP2
3V
AgRP only
c-Fos /AgRP 22.8%
AgRP only 77.2%
b
Bregma -1.46 mm
-1.70 mm
-2.18mm
3V
DMH
VMH
ARC
Depolarized
Non responsive
c
SHLP2
10mV
30s
d
RMP (mV)
aCSF
SHLP2
e
POMC-Cre
POMC
Cre
x
R26-hM4Di F/F
CAG
stop
hM4Di
pta
mCitrine
hM4Di POMC-Cre
f
hM4Di POMC-Cre
DIC
Merge
mCitrine
g
CNO 10μM
-57 mV
10mV
10s
h
RMP (mV)
0.0260
aCSF
CNO
i
Firing rate (Hz)
0.0009
j
Food intake for 4h (g)
0.0013
hM4Di F/F
hM4Di POMC-Cre
CNO
SHLP2
k
ΔBody weight (%) (% of initial value)

Supplementary Fig. 5: Effect of SHLP2 on POMC and AgRP neurons.

a, Percentage of c-Fos activation in AgRP neurons after an ICV administration of SHLP2 (n = 3). Scale bars, 20μm. **b**, Diagram showing the location and responses of recorded POMC neurons across the mediobasal hypothalamus. **c-d,** Representative trace (**c**) of AgRP neurons and effects on resting membrane potential (RMP) (**d**) of a subset of AgRP neurons. **e**, Schematic strategy for recombinant transgenes to generate $hM4Di^{POMC\text{-}Cre}$ male mice. **f**, Fluorescent and DIC figures of targeted POMC neurons expressing the Cre-dependent modified form of the hM4Di receptor in fusion with the fluorescence protein mCitrine. The arrows indicate the targeted cell (n = 6 from 2 animals). **g-i**, Representative inhibition of POMC neuron (**g**) by CNO application. CNO induced hyperpolarization of POMC neurons expressing hM4Di, showing through decreases in the resting membrane potentials (**h**) and firing rates (**i**) (n = 6 from 2 animals). **j-k,** Cumulative food intake (**j**) and Δbody weight (%) (**k**) in $hM4Di^{POMC\text{-}Cre}$ male mice after ICV SHLP2 administration with CNO injection (n = 10 each $hM4Di^{F/F}$ group, n = 5 each $hM4Di^{POMC\text{-}Cre}$ group). Scale bars, 5μm. n indicates the number of biologically independent mice examined. Data were presented as mean ± SEM. Two-tailed Student's t-tests were used. Wilcoxon matched-pairs signed rank test were used in electrophysiological result. CAG, CMV immediate enhancer/β-actin promoter; hM4Di, Gi-coupled hM4D DREADD; pta, porcine Teschovirus cleavage site; mCitrine; citrine fluorescence. Source data are provided as a Source Data file.

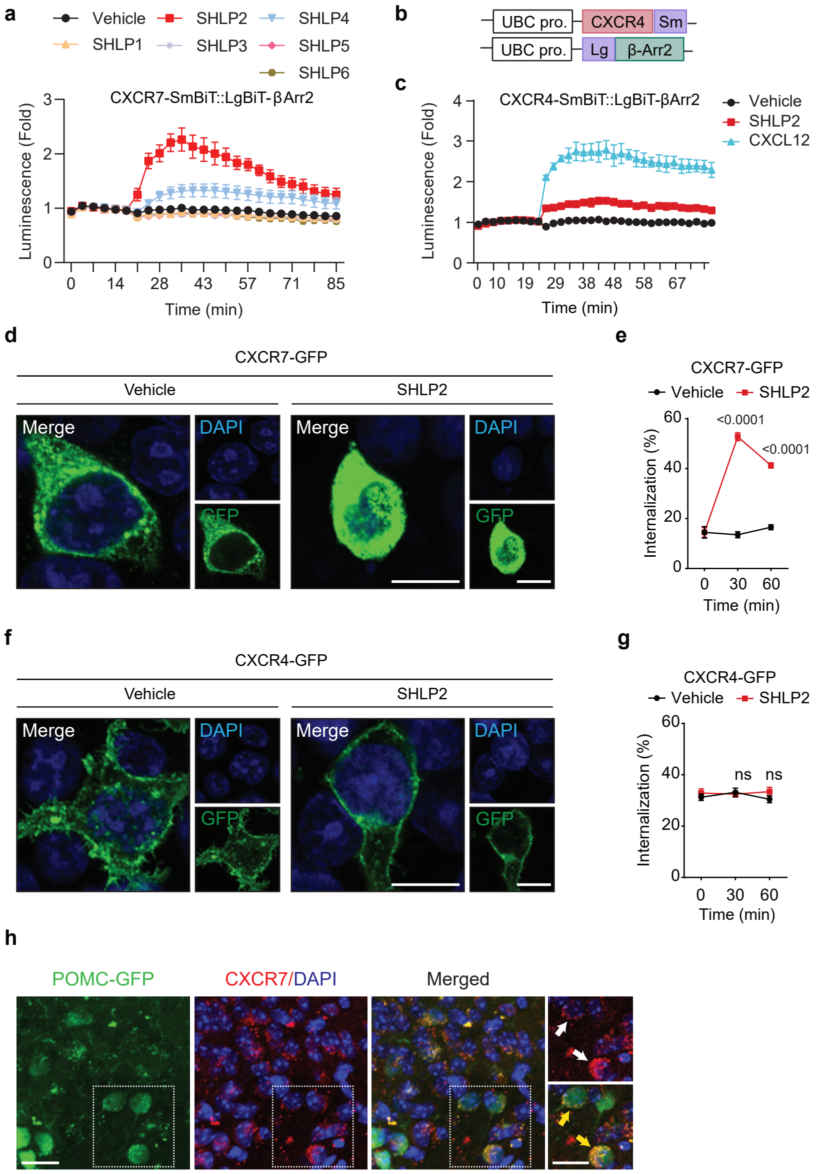

a
Vehicle
SHLP1
SHLP2
SHLP3
SHLP4
SHLP5
SHLP6
CXCR7-SmBiT::LgBiT-βArr2
Luminescence (Fold)
Time (min)
b
UBC pro.
CXCR4
Sm
Lg
β-Arr2
c
CXCR4-SmBiT::LgBiT-βArr2
Vehicle
SHLP2
CXCL12
Luminescence (Fold)
Time (min)
d
CXCR7-GFP
Vehicle
SHLP2
Merge
DAPI
GFP
e
CXCR7-GFP
Vehicle
SHLP2
<0.0001
<0.0001
Internalization (%)
Time (min)
f
CXCR4-GFP
Vehicle
SHLP2
Merge
DAPI
GFP
g
CXCR4-GFP
Vehicle
SHLP2
ns
ns
Internalization (%)
Time (min)
h
POMC-GFP
CXCR7/DAPI
Merged

Supplementary Fig. 6: SHLP2 interacts specifically with CXCR7, but not CXCR4.

a, The β-Arrestin2 recruitment to CXCR7 by SHLP 1 to 6 (*n* = 4 each group). Note that the only SHLP2 exhibited specific activity. **b**, The DNA constructs used to for the NanoLuc complementation-based β-Arrestin2 recruitment to the chemokine receptor CXCR4. **c**, Representative result of β-Arrestin2 recruitment assay to CXCR4 (*n* = 3 saline, *n* = 3 CXCL12, *n* = 4 SHLP2). Note that there is no notable activity by SHLP2 **d,** Representative immunofluorescent staining of CXCR7 upon the application of SHLP2. **e**, The percentage of internalized receptors CXCR7 at different time points after the treatment of SHLP2 (*n* = 3 each group). **f**, Representative immunofluorescent staining of CXCR4 upon the application of SHLP2. **g**, The percentage of internalized receptors CXCR4 at different time points after the treatment of SHLP2 (*n* = 3 each group). Scale bars, 10µm. **h**, Representative images of CXCR7 expression in POMC neurons (*n* = 3 each group). Scale bars, 50µm. *n* indicates the number of biologically independent experimental groups examined. Data were presented as mean ± SD. ns: no significance by two-way ANOVA followed by Sidak multiple comparison tests (**e and g**). Source data are provided as a Source Data file.

Supplementary Table 1. The clinical characteristics of the sutdy subjects

	Healthy (n= 7)	Obese (n= 6)	Diabetic (n= 7)
Age (year)	55.1± 14.9	41.3± 23.7	42.3± 18.7
BMI (kg/m^2)	22.6± 5.1	31.9± 2.9	31.4± 2.3
TG (mg/dL)	85.4± 101.8	192.2± 164.8	174.4± 190.6
Cholesterol (mg/dL)	195.3± 43.7	217.2± 32.2	202.3± 28.7
AST (U/L)	24.9± 9.1	36.8± 21.2	34.4± 18.6
ALT (IU/L)	22.7± 19.3	60.6± 42.4	62.0± 39
Gamma-GT (U/L)	14.7± 11.3	46.8± 45.2	57.9± 61.1

BMI, Body mass index; TG, Triglyceride; AST, Aspartate aminotransferase; ALT, Alanine aminotransferase; Gamma-GT, Gamma-glutamyl transferase

Supplementary Table 2. Primer sequences used for RT-PCR analyses

**Gene**	Forward primer (5’-3’)	Reverse primer (5’-3’)
Ucp1	GGCAACAAGAGCTGACAGTA	GGCCCTTGTAAACAACAAAA
Cox2	ATAACCGAGTCGTTCTGCCAAT	TTTCAGAGCATTGGCCATAGAA
Rps18	TGTGTTAGGGGACTGGTGGACA	CATCACCCACTTACCCCCAAAA
Pomc	CAGGTCCTGGAGTCCGAC	CATGAAGCCACCGTAACG
Npy	CTACTCCGCTCTGCGACACT	AGTGTCTCAGGGCTGGATCTC
Agrp	CGGCCACGAACCTCTGTAG	CTCATCCCCTGCCTTTGC
Cart	AGAAGAAGTACGGCCAAGTC	GGACAGTCACACAGCTTCC
Pgc1α	AACCACACCCACAGGATCAGA	TCTTCGCTTTATTGCTCCATGA
Dio2	TTCTCCAACTGCCTCTTCCTG	CCCATCAGCGGTCTTCTCC
Pparγ	CAAGAATACCAAAGTGCGATCAA	GAGCTGGGTCTTTTCAGAATAATAAG
Srebp-1c	GGAGCCATGGATTGCACATT	GGCCCGGGAAGTCACTGT
Fasn	GGTGTGGTGGGTTTGGTGAATTGT	TCACGAGGTCATGCTTTAGCACCT
Hprt	CTCATGGACTGATTATGGACAGGAC	GCAGGTCAGCAAAGAACTTATAGCC
Scd1	AGTGCAGCAGGACCATGAGAATGA	TCCCTCCGGAAATGAACGAGAGAA
Acaca	AGGAAGATGGTGTCCCGCTCTG	GGGGAGATGTGCTGGGTCAT
Fabp4	AAGAGAAAACGAGATGGTGACAA	CTTGTGGAAGTCACGCCTTT
Prdm16	CGTCCACACGGAAGAGCGTGA	TGGAGGTTGCTGGGGTCCGT
Nrf1	CGATGGGATTCCAGTCTCTGT	TGAGCATCTCTGGGATAAATGC

Supplementary Table 3. Sequences of peptides

Peptide name	Sequence
Small humanin-like peptide 1 (SHLP1)	MCHWAGGASNTGDARGDVFGKQAG
Small humanin-like peptide 2 (SHLP2)	MGVKFFTLSTRFFPSVQRAVPLWTNS
Small humanin-like peptide 3 (SHLP3)	MLGYNFSSFPCGTISIAPGFNFYRLYFIWVNGLAKVVW
Small humanin-like peptide 4 (SHLP4)	MLEVMFLVNRRGKICRVPFTFFNLSL
Small humanin-like peptide 5 (SHLP5)	MYCSEVGFCSEVAPTEIFNAGLVV
Small humanin-like peptide 6 (SHLP6)	MLDQDIPMVQPLLKVRLFND
Scrambled peptide	MKSFRVTVRPASLTSGVFTNLQFPWF
