# Supplementary material for: Mitochondria-derived peptide SHLP2 regulates energy homeostasis through the activation of hypothalamic neurons
Source: Nat Commun. 2023 Jul 19;14:4321. doi: 10.1038/s41467-023-40082-7 (PMC10356901; doi:10.1038/s41467-023-40082-7)
Supplement: Supplementary file 3 — Reporting Summary [file 41467_2023_40082_MOESM3_ESM.pdf]

nature portfolio

Corresponding author(s): Ki Woo Kim

Last updated by author(s): Jun 20, 2023

Reporting Summary

Nature Portfolio wishes to improve the reproducibility of the work that we publish. This form provides structure for consistency and transparency in reporting. For further information on Nature Portfolio policies, see our Editorial Policies and the Editorial Policy Checklist.

Statistics

For all statistical analyses, confirm that the following items are present in the figure legend, table legend, main text, or Methods section.

n/a	Confirmed	
☐	☒	The exact sample size (*n*) for each experimental group/condition, given as a discrete number and unit of measurement
☐	☒	A statement on whether measurements were taken from distinct samples or whether the same sample was measured repeatedly
☐	☒	The statistical test(s) used AND whether they are one- or two-sided<br>*Only common tests should be described solely by name; describe more complex techniques in the Methods section.*
☐	☒	A description of all covariates tested
☐	☒	A description of any assumptions or corrections, such as tests of normality and adjustment for multiple comparisons
☐	☒	A full description of the statistical parameters including central tendency (e.g. means) or other basic estimates (e.g. regression coefficient) AND variation (e.g. standard deviation) or associated estimates of uncertainty (e.g. confidence intervals)
☐	☒	For null hypothesis testing, the test statistic (e.g. *F*, *t*, *r*) with confidence intervals, effect sizes, degrees of freedom and *P* value noted<br>*Give P values as exact values whenever suitable.*
☒	☐	For Bayesian analysis, information on the choice of priors and Markov chain Monte Carlo settings
☒	☐	For hierarchical and complex designs, identification of the appropriate level for tests and full reporting of outcomes
☒	☐	Estimates of effect sizes (e.g. Cohen's *d*, Pearson's *r*), indicating how they were calculated

Our web collection on statistics for biologists contains articles on many of the points above.

Software and code

Policy information about availability of computer code

Data collection	Carl Zeiss Zen version 2.6 imaging software. Victor Nivo Multimode Plate reader. 12-channel PhenoMaster Home Cage System. Thermalert TH-5 thermometer. Dual-energy X-ray absorptiometry (DEXA). BioRad CFX Real-Time PCR system. Clampfit version 10 software.
Data analysis	Fiji (image J2) version 2.3.0 or image J version 2.3.0. GraphPadPrism, version 8.0; Statistical analysis and graphing software. Adobe Photoshop (CS6, ver 13.0 x64).

For manuscripts utilizing custom algorithms or software that are central to the research but not yet described in published literature, software must be made available to editors and reviewers. We strongly encourage code deposition in a community repository (e.g. GitHub). See the Nature Portfolio guidelines for submitting code & software for further information.

Data

Policy information about availability of data

All manuscripts must include a data availability statement. This statement should provide the following information, where applicable:

- Accession codes, unique identifiers, or web links for publicly available datasets
- A description of any restrictions on data availability
- For clinical datasets or third party data, please ensure that the statement adheres to our policy

The datasets generated in this study are provided in the Supplementary Information /Source Data file. Supplementary Information/ Source data are provided with this paper.

Research involving human participants, their data, or biological material

Policy information about studies with human participants or human data. See also policy information about sex, gender (identity/presentation), and sexual orientation and race, ethnicity and racism.

Reporting on sex and gender	Blood samples prepared for immuno dot blot assay were from healthy individuals or from obese and diabetic patients aged 20-70 years old. we used only male samples due to the unavailability of enough female healthy controls
Reporting on race, ethnicity, or other socially relevant groupings	No self-selection or group allocation biases impact the results to our knowledge.
Population characteristics	Population characteristics in supplementary table 1<br>BMI (body mass index): 18.1-34.8 kg/m2,<br>TG (Triglyceride): 48-365 mg/dL,<br>Cholesterol: 160-284mg/dL,<br>AST (aspartate aminotransferase): 16-58U/L,<br>ALT (alanine aminotransferase): 11-103IU/L,<br>Gamma-GT: 11-119U/L.
Recruitment	Patients were consecutively recruited and samples obtained from healthy or from obese or diabetic patients as stated in Methods section. No self-selection or group allocation biases impact the results to our knowledge.
Ethics oversight	All participants provided written informed consent and the Ethics Committee of the Yonsei University College of Medicine approved this study (4-2017-1168), which conforms to the ethical principles of the 1975 Declaration of Helsinki.

Note that full information on the approval of the study protocol must also be provided in the manuscript.

Field-specific reporting

Please select the one below that is the best fit for your research. If you are not sure, read the appropriate sections before making your selection.

☒ Life sciences ☐ Behavioural & social sciences ☐ Ecological, evolutionary & environmental sciences

For a reference copy of the document with all sections, see nature.com/documents/nr-reporting-summary-flat.pdf

Life sciences study design

All studies must disclose on these points even when the disclosure is negative.

Sample size	Sample size is indicated in the figure legend in each experiment. Cell numbers of relative experiments in this study were determined based on our experience and previous studies in this field. All experiments were taken from at least 4 samples (biological replicates) with similar results.
Data exclusions	No data were excluded from the analyses.
Replication	All experiments were repeated at least three times unless otherwise indicated, and all attempts at replication were successful
Randomization	Samples were randomly assigned to experiments.
Blinding	Investigators were blinded to group allocation during data collection and analysis when possible.

Reporting for specific materials, systems and methods

We require information from authors about some types of materials, experimental systems and methods used in many studies. Here, indicate whether each material, system or method listed is relevant to your study. If you are not sure if a list item applies to your research, read the appropriate section before selecting a response.

Materials & experimental systems

n/a	Involved in the study
☐	☒ Antibodies
☐	☒ Eukaryotic cell lines
☒	☐ Palaeontology and archaeology
☐	☒ Animals and other organisms
☒	☐ Clinical data
☒	☐ Dual use research of concern
☒	☐ Plants

Methods

n/a	Involved in the study
☒	☐ ChIP-seq
☒	☐ Flow cytometry
☒	☐ MRI-based neuroimaging

Antibodies

Antibodies used

Primary Antibodies:
Anti-SHLP2 (Ab Frontier, http://www.younginfrontier.com/laboratory/abfrontier/): newly generated
α-tubulin (Developmental Studies Hybridoma Bank, 12G10), dilution 1:5000
UCP1 primary antibody (Abcam, ab10983), dilution 1:1000
p-p38 MAPK (Cell Signaling, 4511), dilution 1:1000
p-ERK (Cell Signaling, 9101), dilution 1:1000
ERK (Cell Signaling, 9102), dilution 1:1000
p-CREB (Cell Signaling, 9196), dilution 1:1000
c-Fos antibody (Cell Signaling, 2250), dilution 1:1000
GFP (Sigma-Aldrich, G1544), dilution 1:3000
DsRed antibody (TaKaRa, 632496), dilution 1:200
CXCR7 antibody (Proteintech, 20423-1-AP), dilution 1:500

Secondary Antibodies:
anti-rabbit secondary antibody with Alexa Fluor 488 (Invitrogen, A21206), dilution 1:1000
anti-rabbit IgG (Invitrogen, 31460), dilution 1:3000
anti-mouse IgG (Invitrogen, 62-6520), dilution 1:2000
biotinylated donkey anti-rabbit IgG (Cat. No. 065-152, Jackson ImmnuoResearch), dilution 1:1000
Alexa Fluor 594 (Invitrogen, A11012), dilution 1:1000

Validation

All antibodies used were purchased or generated from commercial vendors.
All antibodies have been validated according to the supplier's data sheets.

Primary Antibodies:
αα-tubulin (Developmental Studies Hybridoma Bank, 12G10):https://www.citeab.com/antibodies/149755-12g10-anti-alpha-tubulin
UCP1 primary antibody (Abcam, ab10983):https://www.abcam.com/products/primary-antibodies/ucp1-antibody-ab10983.html
p-p38 MAPK (Cell Signaling, 4511): https://www.cellsignal.com/products/primary-antibodies/phospho-p38-mapk-thr180-tyr182-d3f9-xp-rabbit-mab/4511
p-ERK (Cell Signaling, 9101):https://www.cellsignal.com/products/primary-antibodies/phospho-p44-42-mapk-erk1-2-thr202-tyr204-antibody/9101
ERK (Cell Signaling, 9102):https://www.cellsignal.com/products/primary-antibodies/p44-42-mapk-erk1-2-antibody/9102
p-CREB (Cell Signaling, 9196): https://www.cellsignal.com/products/primary-antibodies/phospho-creb-ser133-1b6-mouse-mab/9196
c-Fos antibody (Cell Signaling, 2250):https://www.cellsignal.com/products/primary-antibodies/c-fos-9f6-rabbit-mab/2250
GFP (Sigma-Aldrich, G1544): https://www.sigmaaldrich.com/KR/ko/product/sigma/g1544
DsRed antibody (TaKaRa, 632496):https://www.takarabio.com/products/antibodies-and-elisa/fluorescent-protein-antibodies/red-fluorescent-protein-antibodies
CXCR7 antibody (Proteintech, 20423-1-AP): https://www.ptglab.com/products/CXCR7-Antibody-20423-1-AP.htm

Secondary Antibodies:
anti-rabbit secondary antibody with Alexa Fluor 488 (Invitrogen, A21206): https://www.thermofisher.com/antibody/product/Donkey-nti-rabbit IgG (Invitrogen, 31460), : anti-Rabbit-IgG-H-L-Highly-Cross-Adsorbed-Secondary-Antibody-Polyclonal/A-21206
https://www.thermofisher.com/antibody/product/Goat-anti-Rabbit-IgG-H-L-Secondary-Antibody-Polyclonal/31460
anti-mouse IgG (Invitrogen, 62-6520): https://www.thermofisher.com/order/genome-database/generatePdf?productName=Mouse%20IgG%20(H+L)&assayType=PRANT&detailed=true&productId=62-6520
biotinylated donkey anti-rabbit IgG (Cat. No. 711-065-152, Jackson ImmnuoResearch):https://www.jacksonimmuno.com/catalog/products/711-065-152
Alexa Fluor 594 (Invitrogen, A11012): https://www.thermofisher.com/antibody/product/Goat-anti-Rabbit-IgG-H-L-Cross-Adsorbed-Secondary-Antibody-Polyclonal/A-11012

We also validated and determined the appropriate concentrations before using in our experiments.
Anti-SHLP2 were generated from Ab Frontier and Validation information can be found on manuscript (Supplementary Fig. 1 a-d).

Eukaryotic cell lines

Policy information about cell lines and Sex and Gender in Research

Cell line source(s)	HEK 293T (CRL-3216), CHO-K1 (CCL-61) were obtained from American Type Culture Collection (ATCC).
Authentication	None of the cell lines have been authenticated.
Mycoplasma contamination	All cell lines tested negative for mycoplasma contamination.
Commonly misidentified lines (See ICLAC register)	No commonly misidentified cell lines were used in the study.

Animals and other research organisms

Policy information about studies involving animals; ARRIVE guidelines recommended for reporting animal research, and Sex and Gender in Research

Laboratory animals	C57BL/6J mice (8-12 weeks) (JAX No. 000664)<br>POMC-Cre (JAX No. 005965)<br>R26-tdTomato (JAX No. 007914)<br>R26-hM4Di/ mCitrine (JAX No. 026219)<br>AgRP-Ires-Cre (JAX No. 012899)<br>C57BL/6J-ob/ob (SLC, Inc. Tokyo, Japan), Hamamatsu University School of Medicine Institute for Experimental Animals, C57 background<br>C57BLKS/J-db/db (JAX No. 000662)<br>New Zealand white rabbits of 8 weeks age (1.8~2.0 kg), obtained from the Sinyang<br><br>Detailed gender and age information of mice were specified for each experiment in the manuscript.<br><br>All animals were housed under a controlled temperature 22 ± 1°C and humidity 55 %. Mice were fed with either regular chow diet (LabDiet, 5053)<br>or high fat diet (Research Diets, D12492) and provided with water ad libitum. Male mice, aged 8 to 12 weeks, were used.
Wild animals	No wild animals were used.
Reporting on sex	Only adult male mice were used in our experiments. In this study, we investigated the functional role of SHLP2 in controlling obesity and related metabolic diseases. Male mice are more prone to developing metabolic dysfunction than female mice under HFD feeding conditions [PMID: 34599964]. Therefore, we conducted our study on male mice. We have included information about the gender in the Abstract section of our revised manuscript.
Field-collected samples	This study did not involve field-collected samples.
Ethics oversight	All animal experiments were approved by the Institutional Animal Care and Use Committee (IACUC) of the Avison Biomedical Research Center, Yonsei University.

Note that full information on the approval of the study protocol must also be provided in the manuscript.
